# *Fusarium pseudograminearum* Isolates Show Enhanced Growth and Na^+^ Uptake but Suppressed Mycotoxin Production After Exposure to NaCl at Different Temperatures

**DOI:** 10.3390/biology15030280

**Published:** 2026-02-04

**Authors:** Emiliano Delli Compagni, Mario Masiello, Miriam Haidukowski, Giulia Carmassi, Antonio Moretti, Alberto Pardossi, Susanna Pecchia

**Affiliations:** 1 Department of Agriculture Food and Environment, University of Pisa, Via del Borghetto 80, 56124 Pisa, Italy; giulia.carmassi@unipi.it (G.C.); alberto.pardossi@unipi.it (A.P.); 2 Institute of Sciences of Food Production, National Research Council—CNR-ISPA, Via Amendola 122/O, 70126 Bari, Italy; mario.masiello@cnr.it (M.M.); edithmiriam.haidukowski@cnr.it (M.H.); antonio.moretti@cnr.it (A.M.); 3 Interdepartmental Research Center Nutrafood “Nutraceuticals and Food for Health”, University of Pisa, Via del Borghetto 80, 56124 Pisa, Italy

**Keywords:** deoxynivalenol, *Fusarium* mycotoxins, halophilic fungi, plant diseases, plant pathogens, potassium, *Salicornia europaea*, salinity, sodium, zearalenone

## Abstract

Several *Fusarium* species have demonstrated the ability to thrive in saline soils and to tolerate or prefer high salt concentrations. In this context, the potential halophilic or halotolerant lifestyle of *Fusarium pseudograminearum* was investigated. Four isolates (3B, PVS-Fu 7, ColPat-1, and CBS 109956) were grown at different temperatures (10, 15, 20, 25, 30, and 35 °C) and NaCl concentrations (0, 7, 14, 21, and 28 g L^−1^), and daily growth, mycotoxin production, and K^+^ and Na^+^ accumulation within hyphae were assessed. All *F. pseudograminearum* isolates exhibited strong adaptability to saline conditions, with significantly enhanced growth in the presence of NaCl. All isolates accumulated Na^+^ within their hyphae while retaining K^+^. The production of deoxynivalenol (DON) and zearalenone (ZEA) was generally suppressed following NaCl exposure, consistent with the known inhibitory effect of reduced water activity on mycotoxin biosynthesis. However, at 22 °C, two isolates, 3B and CBS 109956, showed no significant differences in ZEA production between the control (no salt) and the medium containing the lowest NaCl concentration tested (7 g L^−1^). Notably, isolate 3B, obtained from the halophyte *Salicornia europaea*, retained the highest levels of both Na^+^ and K^+^ within hyphae and showed the greatest overall adaptation to salinity. These results confirm the hypothesis that the ability of *F. pseudograminearum* to colonize and infect a halophytic host is indicative of a halophilic lifestyle. In the context of increasing soil salinization, these findings help identify conditions that permit pathogen persistence without hazardous mycotoxin accumulation.

## Simple Summary

In the context of increasing global environmental changes, understanding the adaptive mechanisms of plant pathogens is crucial for mitigating their impact on agriculture and natural ecosystems. This study tested the hypothesis that the ability of a fungal pathogen to infect a halophytic plant may indicate a halophilic or halotolerant lifestyle. To investigate this, the physiological responses of *Fusarium pseudograminearum* isolate 3B, originally isolated from the halophyte *Salicornia europaea*, were compared with other *F. pseudograminearum* isolates under different NaCl concentrations (0, 7, 14, 21, and 28 g L^−1^) and temperatures (10, 15, 20, 25, 30, and 35 °C). The experimental design included the assessment of (i) daily growth rate on PDA medium, (ii) mycotoxin production, and (iii) hyphal accumulation of K^+^ and Na^+^. The results demonstrated that isolate 3B exhibits physiological traits consistent with halophily. Overall, these findings suggest that adaptation to saline environments may play a role in the ecological success and pathogenic potential of *F. pseudograminearum*.

## 1. Introduction

*Fusarium pseudograminearum* O’Donnell & T. Aoki (syns. *Fusarium graminearum* Group 1; *Gibberella coronicola* T. Aoki & O’Donnell) is a major phytopathogenic fungus responsible for Fusarium crown rot of cereals, a severe and ubiquitous disease that results in considerable reductions in crop yields [1,2]. This fungus is prevalent in many arid and semi-arid regions and can infect a broad range of cereal crops. In addition to its pathogenicity, *F. pseudograminearum* can produce mycotoxins, secondary metabolites that contaminate food and feed, posing serious health risks for humans and animals [3,4]. The most commonly occurring *F. pseudograminearum* mycotoxins include zearalenone (ZEA) and type B trichothecenes.

Zearalenone is a non-steroidal estrogenic mycotoxin structurally similar to natural estrogens, enabling it to bind to estrogenic receptors and interfere with hormonal regulation [5]. In animals, exposure to ZEA has been associated with reproductive disorders, including infertility, altered estrous cycles, and developmental abnormalities [6]. In pigs, a species susceptible to estrogenic compounds, ZEA exposure has been linked to symptoms such as vulvar swelling, reduced fertility, and abortion [7,8]. Other livestock, including cattle and poultry, are also susceptible to its effects [9]. In humans, ZEA’s estrogenic activity raises concerns in children and pregnant women [5,10]. Chronic exposure to ZEA has been suggested to contribute to the development of hormone-dependent cancers [11]. Zearalenone contamination is most associated with maize, but it is also frequently detected in other cereals such as wheat, barley, and oats [8].

Type B trichothecenes, such as deoxynivalenol (DON) and its acetylated derivatives 3-acetyldeoxynivalenol (3ADON) and 15-acetyldeoxynivalenol (15ADON), are a group of sesquiterpenoid mycotoxins. These compounds exert their toxic effects by binding to the ribosomal peptidyl transferase center, thereby inhibiting protein synthesis and disrupting translation in eukaryotes. This interference activates cellular stress responses, including MAPK signaling pathways and apoptosis [12,13]. Acute exposure to DON can cause symptoms such as nausea, vomiting, and diarrhea, while chronic exposure has been associated with immunosuppression and reproductive disorders [14].

Due to their significant health risks, regulatory authorities have established maximum permitted levels for DON and ZEA in food and feed. In the European Union, the maximum level of ZEA in unprocessed maize is 350 µg kg^−1^ and 20 µg kg^−1^ in processed cereal intended for baby diet [15]. For DON, maximum limits range from 1000 µg kg^−1^ in unprocessed cereals to 150 µg kg^−1^ in processed cereal-based infant foods [16].

The management of *Fusarium* mycotoxins requires an integrated approach combining preventive and mitigation strategies. Agronomic practices such as crop rotation, selection of resistant cultivars, and the application of appropriate post-harvest technologies can significantly reduce *Fusarium* infection and subsequent mycotoxin contamination [17,18,19]. Environmental conditions, particularly temperature and humidity, also play a crucial role in mycotoxin biosynthesis. For instance, the production of DON is favored by temperatures between 20 and 30 °C and high water activity (a_w_; >0.97). However, there is considerable inter- and intraspecific variability in response to these factors [20,21,22,23,24,25]. Similarly, the production of ZEA is strongly influenced by temperature and humidity. At 100% relative humidity, ZEA contamination was significantly higher at 20 and 25 °C compared to 30 °C [26]. In contrast, ZEA synthesis was significantly reduced at 15 °C, even under high moisture conditions [27].

Extreme environmental conditions tend to suppress mycotoxin biosynthesis. The production of both DON and ZEA decreased significantly at temperatures exceeding 35 °C [20,21]. Given the strong influence of environmental conditions on mycotoxin biosynthesis, there is a clear need for tailored agricultural and storage strategies to mitigate the risks associated with DON and ZEA contamination. Maintaining grain moisture levels below critical a_w_ thresholds (typically <0.70) and controlling ambient temperatures are essential to inhibit fungal growth and toxin production [20,28].

Recently, a pathogenic and mycotoxigenic strain of *F. pseudograminearum* (3B) was isolated from *Salicornia europaea* L. [29], a halophytic plant that thrives in salt-affected soils and is used in saline agriculture and for soil remediation [30]. The presence of *Fusarium* spp. in saline environments has been well documented, and several studies have reported halophilic or halotolerant traits in several *Fusarium* species [31,32,33,34]. Recently, two populations of *F. pseudograminearum*, representing the 3AcDON and 15AcDON chemotypes, showed a strong tolerance to osmotic stress (1M sorbitol) and salt stress (1M NaCl) [35].

Based on this evidence, the hypothesis formulated in this study is that the ability to infect a halophytic host may imply a halophilic or halotolerant lifestyle in *F. pseudograminearum* according to the definition of Yovchevska et al. [36]. To test this, the physiological responses of *F. pseudograminearum* 3B, alongside other *F. pseudograminearum* isolates provided by other laboratories in Europe, were evaluated at different NaCl concentrations (0, 7, 14, 21, and 28 g L^−1^) and temperatures (10, 15, 20, 25, 30, and 35 °C). The parameters assessed included daily growth rate, ZEA and DON production, and the concentration of cations (K^+^ and Na^+^) in fungal hyphae. To our knowledge, this is the first report on the relationship between salt concentrations, temperatures, and mycotoxin production in the phytopathogenic fungus *F. pseudograminearum*.

The expected results could offer valuable insights into both growth and mycotoxin production dynamics when assessing the ecological and pathological implications of salinity in crop systems.

## 2. Materials and Methods

### 2.1. Fungal Isolates: Origin and Storage Conditions

Four *F. pseudograminearum* isolates were used in this study. Isolate 3B was obtained from the halophytic plant *S. europaea* [29], while isolates PVS-Fu 7 and ColPat-1 were previously recovered from wheat fields in Italy and Spain, respectively [37,38]. Isolate CBS 109955, a holotype of *F. pseudograminearum*, was sourced from the Centraalbureau voor Schimmelcultures (Utrecht, the Netherlands); it was originally isolated from wheat crown tissue in Australia. All isolates were cultivated on Potato Dextrose Agar plates (PDA; 42 g L^−1^; BioLife, Milan, Italy) and maintained on Synthetic Nutrient-poor Agar (SNA) [39] for further analyses.

### 2.2. Growth Assays Under Different Salinity and Temperatures (Experiment 1)

To determine the optimal growth conditions and assess salinity and temperature tolerance, the four *F. pseudograminearum* isolates were tested for their ability to grow under varying environmental conditions. The experiment included five biological replicates for each treatment. A 6 mm mycelial plug was excised from the actively growing margin of each colony and transferred onto 9 cm Petri dishes containing PDA with NaCl at concentrations of 0, 7, 14, 21, and 28 g L^−1^, corresponding, respectively, to 0, 119.8, 239.6, 359.3, and 479.1 mM. We aimed to test low, intermediate, high, and very high salinity levels that reflect the ranges typically found in environments where halophytes grow. To ensure a correct solidification of the salt-supplemented media, an additional 0.5% (*w*/*v*) of agar was added. All plates were incubated at 10, 15, 20, 25, 30, and 35 °C in complete darkness. Mycelial plugs were placed at the edge of each plate, and the daily mycelial growth was measured until the colony reached the opposite edge. The growth rate was calculated based on measurements taken every 24 h during the exponential growth phase.

### 2.3. Zearalenone and Deoxynivalenol Production on Barley Grains (Experiment 2)

Mycotoxin production was assessed by growing the *F. pseudograminearum* isolates on moistened barley grains following the protocol described by Delli Compagni et al. [29], with minor modifications.

Briefly, 30 g of dry barley grains were rinsed overnight and then double-autoclaved with a 24 h interval to ensure sterility. For the salt treatments, 25 mL of distilled water containing 0, 7, and 28 g L^−1^ NaCl was added to each flask containing the sterilized grains. The flasks were then inoculated with each fungal isolate and incubated for 21 days in the dark at 22, 25, and 28 °C, selected to represent the optimal temperature (25 °C) and suboptimal temperatures. For each treatment, three biological replicates were considered.

For ZEA determination, 2 g of ground barley grains were mixed with 15 mL of extraction solvent—acetonitrile/water (84:16, *v*/*v*) + 1% of acetic acid—into a 50 mL polypropylene centrifuge tube. The mixture was shaken at 150 rpm for 60 min. After extraction, samples were centrifuged at 14,500 rpm for 15 min. A total of 1 mL of resulting supernatant was collected and dried at 50 °C under a gentle stream of nitrogen. The extracts were reconstituted in 1 mL of acetonitrile/water (10:90, *v*/*v*) and vortex-mixed for 15 s. A 10 µL aliquot of each extract was injected into a UPLC apparatus (Agilent 1290 Series, Agilent Technologies Inc., Santa Clara, CA, USA), with a column heater set at 35 °C and a fluorometric detector (FLD) (λ_ex_ = 274 nm, λ_em_ = 440 nm). The analytical column was a ZORBAX Eclipse Plus C18, 100 mm × 2.1 mm (Agilent Technologies Inc., Santa Clara, CA, USA). The mobile phase was an isocratic composition of water and acetonitrile delivered at a flow rate of 0.25 mL min^−1^ for 5 min. Under these analytical conditions, the retention time of ZEA was approximately 3.3 min. Quantification was performed by comparing peak areas with calibration curves generated from standard solutions. When necessary, extracts were appropriately diluted to fall within the linear range of detection. The limit of detection (LOD) for ZEA quantification was 0.1 mg kg^−1^ (dry weight) [40].

For the determination of DON, 2 g of ground barley grains were extracted with 15 mL of ultra-pure distilled water (EASYpure^®^ II LF, Thermo Scientific, Waltham, MA, USA; resistivity 18.2 MΩ cm^−1^). The mixture was shaken at 150 rpm for 60 min. Extracts were filtered through a glass microfiber filter Whatman GF/A (Whatman, Maidston, UK) and 10 mL of the filtrate was passed through DONTest immunoaffinity columns (VICAM, Milford, MA, USA) at a flow rate of approximately one drop per second. The columns were then washed with 10 mL of distilled water, and DON was eluted with 1.5 mL of methanol. The cleaned-up extracts were collected in 4 mL screw-cap amber vials and dried at 50 °C under a gentle stream of nitrogen. The dried residues were reconstituted in 200 μL of methanol/water 40:60 (*v*/*v*) containing 5 mM of ammonium acetate. Quantification was performed using a reversed-phase HPLC system (1100 Series HPLC Value System, Agilent Technologies Inc., Santa Clara, CA, USA) equipped with a diode-array UV detector set at 220 nm. The column was a Synergi 4 μm Hydro RP 80A, 150 mm × 3 mm (Phenomenex, Torrance, CA, USA). The mobile phase consisted of acetonitrile/water (10:90) eluted at a flow rate of 0.5 mL min^−1^. Under these conditions, the retention time of DON was about 2.4 min. Quantification was based on peak area comparison with calibration curves prepared from standard solutions. The LOD for DON quantification was 10 µg kg^−1^ (dry weight) [40].

### 2.4. Determination of Cation Levels in Fungal Hyphae (Experiment 3)

To evaluate the accumulation of Na^+^ and K^+^ within fungal hyphae, *F. pseudograminearum* isolates were grown on PDA plates amended with 0, 7, and 28 g L^−1^ NaCl. A 6 mm mycelial plug was taken from the actively growing margin of each colony and placed at the center of a sterilized cellophane disk overlaid on a 9 cm PDA plate. After incubation at 22, 25, and 28 °C for seven days, the mycelium was gently collected by scraping the cellophane disk and dried in a ventilated oven at 50 °C until a constant weight was achieved. To determine cation concentrations within fungal hyphae, dried samples were finely ground using a mortar and pestle. A powdered subsample of 100 mg was extracted with 15 mL of 1N HCl overnight following the method described by Asch et al. [41]. The extract was filtered through Whatman No. 1 filter paper. Potassium and Na^+^ concentrations were quantified using atomic absorption spectrophotometry (AA500FG, PG Instruments, Leicestershire, UK). Additionally, the K^+^/Na^+^ molar ratio was calculated. The experiment included three biological replicates for each treatment.

### 2.5. Statistical Analysis

All data were analyzed using two-way and three-way ANOVA, performed with the *aov* function in RStudio (v. 12.0 + 467) [42]. The normality of the data distribution was assessed using the Shapiro–Wilk test (*Shapiro test* function), while homoscedasticity was evaluated using Levene’s test (*Levene test* function from the *car* package version 3.1-3). Mean comparisons were conducted using Tukey’s Honest Significant Difference (HSD) test at a significance level of *p* ≤ 0.05, implemented via the *Tukey HSD* function. A three-way ANOVA was first conducted to assess the significance of the main effects of isolate, temperature, and salinity, and their interactions, on all measured variables. To facilitate clearer presentation and interpretation of the results, the temperature × salinity interaction was then examined separately within each isolate by performing a two-way ANOVA on each isolate-specific dataset, analyzing the effects of temperature, NaCl concentration, and their interaction. For data related to ZEA and DON production, natural logarithmic transformation (*ln*) was applied to meet ANOVA assumptions. When no mycotoxin production was detected, values were substituted with LOD/2.

## 3. Results

### 3.1. Growth Assays Under Different Salinity and Temperatures

In Experiment 1, the four *F. pseudograminearum* isolates were incubated on PDA plates at six temperature levels (10, 15, 20, 25, 30, and 35 °C) and five NaCl concentrations (0, 7, 14, 21, and 28 g L^−1^). The daily growth rate (mm day^−1^) was recorded during the exponential growth phase. No growth was observed for any isolate at 35 °C.

The results of the three-way analysis of variance are presented in Table S1. The daily growth rates differed significantly among isolates, with 3B (11.0 ± 0.3 mm day^−1^) and PVS-Fu7 (10.9 ± 0.4 mm day^−1^) showing higher growth than isolates ColPat-351 (9.1 ± 0.3 mm day^−1^) and CBS 109956 (7.7 ± 0.1 mm day^−1^). Growth increased significantly with temperature, reaching a maximum at 25 °C (14.0 ± 0.3 mm day^−1^) and decreasing sharply at 10 °C (3.7 ± 0.0 mm day^−1^). Salinity also had a significant effect on growth: the highest rates occurred at 7 and 14 g L^−1^ NaCl (10.4 ± 0.4 and 10.5 ± 0.4 mm day^−1^, respectively), whereas growth was reduced in the absence of NaCl (7.7 ± 0.3 mm day^−1^).

**Figure 1:**
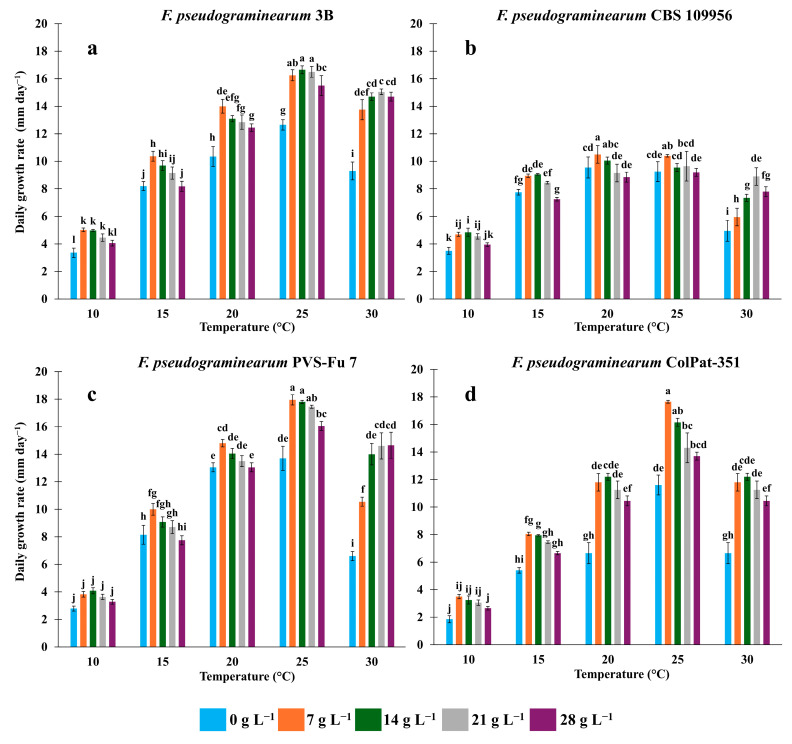
The effect of NaCl salinity and temperature (10, 15, 20, 25, and 30 °C) on the daily growth rate (mm day^−1^) of four *Fusarium pseudograminearum* isolates [3B (**a**), CBS 109956 (**b**), PVS-Fu 7 (**c**), and ColPat-351 (**d**)] grown on Potato Dextrose Agar. The daily growth rate was recorded during the exponential phase. Data were subjected to two-way ANOVA and means (*n* = 5; ±SE) were separated by Tukey’s HSD post hoc test. Different letters indicate statistically significant differences at *p* ≤ 0.001.

Table S2 presents the results of the two-way ANOVA, revealing a highly significant effect (*p* ≤ 0.001) of temperature, salinity, and their interaction on mycelial growth across all *F. pseudograminearum* isolates. The daily growth rates of the four isolates in response to varying salinity and temperature conditions are illustrated in Figure 1. Overall, all *F. pseudograminearum* isolates exhibited similar growth responses. A positive effect of NaCl on mycelial growth was observed, with the highest growth rates recorded at salt concentrations higher than 7 g L^−1^. At each salinity concentration, the lowest growth rates were consistently observed at 10 °C, while optimal growth occurred between 20 and 25 °C.

All isolates demonstrated enhanced growth in response to increasing salinity at higher temperatures, with the most pronounced effects observed at 30 °C. Notably, isolate CBS 109956 showed improved growth at 28 g L^−1^ NaCl only at 30 °C, compared to the control grown without salt. Similarly, isolates PVS-Fu 7 and 3B exhibited optimal growth at salinity levels exceeding 14 g L^−1^ (Figure 1a–c). The latter demonstrated great adaptability to high salinity at temperatures above 20 °C and exhibited better growth in saline media than in salt-free conditions. This trend was also observed in isolate ColPat-351 (Figure 1d): at 28 g L^−1^ NaCl, the daily growth rate was generally reduced but remained significantly higher compared to the control treatment. The highest growth rate was recorded in isolate PVS-Fu 7 at 25 °C, with 17.9 and 17.8 mm day^−1^ observed at 7 and 14 g L^−1^ NaCl, respectively (Figure 1c).

The impact of salinity on mycelial growth was particularly pronounced at 30 °C, where a two-fold increase in growth rate was observed. Mean values for the four isolates increased from 6.6 mm day^−1^ in the salt-free medium to 10.6 (+60%), 14 (+112%), 14.6 (+121%), and 14.7 (+122%) mm day^−1^ at 7, 14, 21, and 28 g L^−1^ NaCl, respectively. For isolate 3B, the highest growth rates were recorded at 25 °C with 14 and 21 g L^−1^ NaCl (16.7 and 16.5 mm day^−1^, respectively). Up to 21 g L^−1^ NaCl, growth rate was significantly higher than in the absence of salt across all tested temperatures (Figure 1a). For isolate ColPat-351, optimal growth rate was observed at 25 °C with 7 g L^−1^ NaCl, reaching 17.6 mm day^−1^ (Figure 1d). In contrast, isolate CBS 109956 showed the slowest growth rate among the four isolates. Its optimum growth rate was recorded at 20 °C with 7 g L^−1^ NaCl, reaching 10.5 and 10.4 mm day^−1^ (Figure 1b).

### 3.2. Zearalenone and Deoxynivalenol Production on Barley Grains

Experiment 1 was instrumental in identifying the optimal and suboptimal temperatures and salinity levels of the *F. pseudograminearum* isolates. Based on these results, in Experiment 2, the following conditions were selected for mycotoxin production assays: temperatures of 22, 25, and 28 °C, and NaCl concentrations of 0, 7, and 28 g L^−1^. Under these conditions, the production of ZEA (expressed as mg kg^−1^) and DON (expressed as µg kg^−1^) was assessed by cultivating the four isolates on moistened barley grains for 21 days. A control (uninoculated barley seeds) was included in the study; however, as no toxin was detected (<LOD) in these samples, results are not shown.

A summary of the three-way ANOVA results is provided in Table S3. ZEA production varied significantly among isolates, with the highest mean value observed in isolate 3B (211.5 ± 68.4 mg kg^−1^) and progressively lower levels in isolates CBS 109956 (55.9 ± 12.9 mg kg^−1^), PVS-Fu7 (35.3 ± 10.2 mg kg^−1^), and ColPat-351 (12.8 ± 3.5 mg kg^−1^). Across temperatures, ZEA production was highest at 22 °C and 25 °C (61.3 ± 10.1 and 170.8 ± 52.7 mg kg^−1^, respectively) and was markedly reduced at 28 °C (4.6 ± 1.1 mg kg^−1^). Increasing NaCl concentration strongly suppressed ZEA production, which declined from 0 g L^−1^ (161.4 ± 51.6 mg kg^−1^) to 7 g L^−1^ (62.5 ± 16.4 mg kg^−1^) and 28 g L^−1^ (12.7 ± 5.8 mg kg^−1^).

Table S4 summarizes the results of the two-way ANOVA for ZEA production. A highly significant effect (*p* ≤ 0.001) of temperature, salinity, and their interaction was observed for all *F. pseudograminearum* isolates, except for isolate 3B. For this isolate, the interaction between temperature and salinity was slightly less pronounced but still statistically significant (*p* ≤ 0.01).

All *F. pseudograminearum* isolates were able to produce ZEA under all considered conditions (Table 1). The presence of NaCl consistently led to a reduction in ZEA biosynthesis, with higher production observed in salt-free media. The optimal temperature for ZEA production ranged between 22 °C and 25 °C. Isolates 3B and CBS 109956 reached a production peak at 25 °C, while isolate PVS-Fu 7 showed maximum output at both 22 and 25 °C. Isolate ColPat-351 was most efficient at 22 °C. A significant decrease in ZEA production was observed across all *F. pseudograminearum* isolates at 28 °C, with reduction rates ranging from 80% to 99% compared to their respective optimal conditions. Under the most severe condition, 28 °C combined with 28 g L^−1^ NaCl, ZEA production was the lowest, though still detectable at levels above 1 mg kg^−1^.

The highest ZEA concentration was recorded in isolate 3B, which produced over 1000 mg kg^−1^ at 25 °C in the absence of salt. This isolate also maintained relatively high production levels at 7 and 28 g L^−1^ NaCl. Interestingly, in isolate 3B, ZEA production at 28 g L^−1^ NaCl was still comparable to that observed in the salt-free medium at 22 °C, suggesting a stronger dependence on temperature than on salinity. This highlights a narrow optimal temperature range for ZEA biosynthesis in this isolate. Additionally, isolates 3B and CBS 109956 showed no significant reduction in ZEA production at 22 °C when exposed to low NaCl concentrations, despite this not being their optimal temperature.

On the other hand, isolates PVS-Fu 7 and ColPat-351 exhibited greater sensitivity to salinity, consistently producing higher levels of ZEA in the absence of NaCl across all tested temperatures (Table 1). Notably, even under optimal conditions, isolate ColPat-351 yielded the lowest amount of ZEA among the four isolates.

Only isolates 3B and CBS 109956 were able to produce DON (>LOD) under the selected conditions.

The results of the three-way analysis of variance are presented in Table S5. For DON, mean production was substantially higher in isolate 3B (270.3 ± 104.5 µg kg^−1^) than in isolate CBS 109956 (18.4 ± 5.6 µg kg^−1^). DON levels were also highest at 25 °C (408.2 ± 147.5 µg kg^−1^), lower at 22 °C (19.9 ± 5.4 µg kg^−1^), and <LOD at 28 °C. Similarly, DON production decreased with increasing salinity, declining from 0 g L^−1^ (303.2 ± 145 µg kg^−1^) to 7 g L^−1^ (124.9 ± 65.2 µg kg^−1^) and falling below the detection limit at 28 g L^−1^ NaCl.

Two-way ANOVA (Table S6) revealed a significant influence of temperature, salinity, and their interaction on DON production for both isolates 3B and CBS 109956.

Isolate 3B exhibited the highest DON production, exceeding 1000 µg kg^−1^ at 25 °C in the absence of salt (Table 2). DON production significantly declined at the lowest NaCl concentration tested (7 g L^−1^), with a reduction of more than 50% compared to the salt-free condition. At 22 °C, only a small amount of DON was detected and exclusively in the absence of salt.

Isolate CBS 109956 produced low quantities of DON at both 22 °C and 25 °C without NaCl, with no statistically significant differences between these conditions. Salinity had a strong suppressive effect on DON biosynthesis, and no DON was detected at 28 g L^−1^ NaCl for both isolates (Table 2). Like ZEA, 28 °C completely inhibited DON production.

### 3.3. Cation Levels in Fungal Hyphae

In Experiment 3, K^+^ and Na^+^ concentrations within fungal hyphae were determined in *F. pseudograminearum* isolates cultivated on PDA plates for seven days under the same temperature and salinity conditions used for the mycotoxin production assays.

A summary of the three-way ANOVA results is provided in Tables S7–S9. Mean K^+^ concentrations were significantly higher in isolates 3B (15.4 ± 0.3 mg g^−1^) and CBS 109956 (15.8 ± 0.4 mg g^−1^) than in isolates PVS-Fu7 (9.5 ± 0.3 mg g^−1^) and ColPat-351 (8.6 ± 0.2 mg g^−1^). Potassium concentration was not significantly affected by temperature, but it was slightly higher at 28 g L^−1^ NaCl (13.1 ± 0.7 mg g^−1^) than at 0 and 7 g L^−1^ (11.7 and 12.1 mg g^−1^, respectively) (Table S7).

Mean hyphal Na^+^ concentrations were highest in isolate 3B (11.6 ± 1.4 mg g^−1^), intermediate in isolate CBS 109956 (10.5 ± 1.3 mg g^−1^), and lower in isolates PVS-Fu7 (7.7 ± 1.2 mg g^−1^) and ColPat-351 (7.8 ± 1.2 mg g^−1^). Sodium levels increased at 25 °C and 28 °C (averaging 9.6 ± 1.2 mg g^−1^) relative to 22 °C (9.0 ± 1.0 mg g^−1^). As expected, Na^+^ concentration rose sharply with increasing salinity, from 0 g L^−1^ (2.7 ± 0.3 mg g^−1^) to 7 g L^−1^ (7.3 ± 0.1 mg g^−1^) and 28 g L^−1^ NaCl (18.2 ± 0.5 mg g^−1^) (Table S8).

Accordingly, the K^+^/Na^+^ molar ratio differed among isolates, being highest in isolates PVS-Fu7 (3.0 ± 0.8) and ColPat-351 (2.5 ± 0.6) and lowest in isolates 3B (1.1 ± 0.1) and CBS 109956 (1.4 ± 0.2). The ratio increased significantly at 28 °C (3.0 ± 0.7) compared with 22 °C and 25 °C (1.5 ± 0.2) and declined sharply with rising salinity, decreasing from 0 g L^−1^ (4.7 ± 0.6) to 7 g L^−1^ (0.9 ± 0.0) and 28 g L^−1^ NaCl (0.4 ± 0.0) (Table S9).

**Figure 2:**
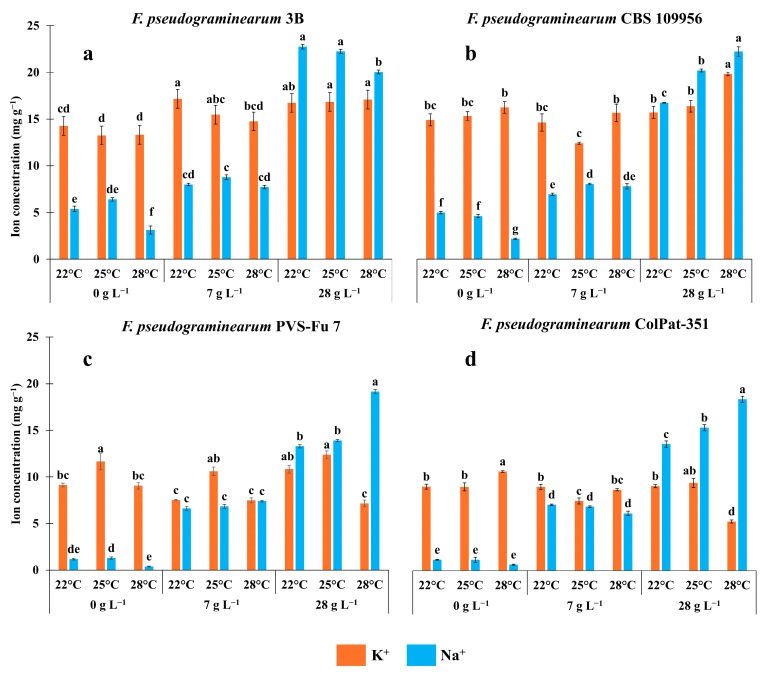
Potassium (K^+^) and sodium (Na^+^) concentration (mg g^−1^ of dry weight) within hyphae of four *Fusarium pseudograminearum* isolates [3B (**a**); CBS 109956 (**b**); PVS-Fu 7 (**c**); and ColPat-351 (**d**)] grown at 22, 25, and 28 °C on Potato Dextrose Agar plates supplemented with 0, 7, and 28 g L^−1^ NaCl for one week. Data were analyzed using two-way ANOVA. Means (*n* = 3; ±SE) were separated by Tukey’s HSD post hoc test. Different letters indicate statistically significant differences at *p* ≤ 0.05, *p* ≤ 0.01 or *p* ≤ 0.001.

Two-way ANOVA revealed that the concentration of both K^+^ and Na^+^ was significantly influenced by temperature, salinity level, and their interaction (Tables S10 and S11). The levels of K^+^ and Na^+^ in fungal hyphae for the four *F. pseudograminearum* isolates are presented in Figure 2.

All isolates demonstrated the ability to accumulate Na^+^ following exposure to NaCl, with a progressive increase in Na^+^ ion accumulation observed at higher NaCl concentrations. Interestingly, a basal level of Na^+^ was detected in hyphae even under salt-free conditions. Isolates 3B and CBS 109956 exhibited relatively high basal Na^+^ concentrations, averaging 5 and 4 mg g^−1^, respectively (Figure 2a,b). In contrast, isolates PVS-Fu 7 and ColPat-351 showed markedly lower levels, both averaging 1 mg g^−1^ (Figure 2c,d). A significant reduction in Na^+^ concentration was observed at 28 °C across all fungal isolates (Figure 2a–d). At a salinity level of 7 g L^−1^ NaCl, isolates PVS-Fu 7 and ColPat-351 exhibited a marked increase in Na^+^ concentration, with no significant differences among temperatures (Figure 2c,d). Specifically, hyphal Na^+^ concentration increased from 1 to 6.7 mg g^−1^ in isolate ColPat-351 and from 0.9 to 6.9 mg g^−1^ in isolate PVS-Fu 7.

All isolates reached their highest Na^+^ concentration at 28 g L^−1^ NaCl. Isolates 3B and CBS 109956 showed the greatest Na^+^ accumulation, averaging 8.2 and 7.7 mg g^−1^, respectively. Under this condition, temperature had a pronounced effect. Isolates CBS 109956, PVS-Fu 7, and ColPat-351 showed a progressive increase in Na^+^ concentration with rising temperatures, reaching a maximum level at 28 °C, with values of 22.4, 19.2, and 18.5 mg g^−1^ respectively (Figure 2b–d). In contrast, isolate 3B showed a reduction in hyphal Na^+^ concentration at 28 °C (Figure 2a). Overall, this isolate recorded the highest Na^+^ accumulation, reaching 22.8 mg g^−1^ at 22 and 25 °C, when cultivated on PDA amended with 28 g L^−1^ NaCl.

The K^+^ concentration within fungal hyphae varied in response to salinity levels and temperatures, with distinct patterns observed among the isolates. Apart from isolate ColPat-351, which showed elevated K^+^ levels at 25 °C under salt-free conditions (Figure 2d), all isolates reached their maximum K^+^ concentrations at the highest NaCl level. However, in isolate PVS-Fu 7, K^+^ accumulation appeared to be more temperature-dependent, with peaks consistently observed at 25 °C across all NaCl concentrations (Figure 2c). In contrast, isolate CBS 109956 exhibited its highest K^+^ concentration at 28 °C (Figure 2b). Interestingly, isolate 3B showed a consistent increase in hyphal K^+^ concentration with rising NaCl levels. At low salinity (7 g L^−1^ NaCl), K^+^ levels were enhanced, though the extent of accumulation varied across temperatures. At high salinity (28 g L^−1^ NaCl), K^+^ peaks were observed across all temperature conditions (Figure 2a).

## 4. Discussion

This study investigated the adaptability of four *F. pseudograminearum* isolates to varying NaCl concentrations and temperatures by evaluating their growth rate, mycotoxin production, and cation (K^+^ and Na^+^) levels within fungal hyphae.

All four *F. pseudograminearum* isolates exhibited a weak halophilic phenotype according to the definition of Yovchevska et al. [36]. Supplementation with NaCl significantly enhanced mycelial growth, with optimal growth rates observed at concentrations between 7 and 14 g L^−1^ (Figure 1a–d). Mycelial growth was influenced by both temperature and salinity, and all isolates displayed markedly faster development at the highest NaCl concentration tested (28 g L^−1^) compared to salt-free conditions. These findings indicate a strong adaptive capacity of *F. pseudograminearum* to saline environments. The cardinal temperature range for growth was 10–30 °C, with optimal development observed between 20 and 25 °C. No growth occurred at 35 °C under any salinity condition, confirming that *F. pseudograminearum* is unable to grow at this temperature on PDA, as previously reported by Singh et al. [43]. Across all isolates, increasing temperatures progressively enhanced halophytism and supported growth at higher NaCl concentrations (Figure 1a–d). This suggests a pattern in which high temperatures amplify the stimulatory effect of NaCl on fungal growth.

The genus *Fusarium* is frequently associated with saline soils and arid environments [31,33]. Recently, a halophilic isolate of *F. solani* showed tolerance to up to 20% NaCl and was capable of growing at 40 °C [34]. Several *Fusarium* species have been shown to tolerate NaCl concentrations up to 12.5%, with optimal growth observed at 2.5% [33]. Additionally, Smolyanyuk et al. [32] reported halotolerance in an isolate member of *Fusarium incarnatum-equiseti* species complex, which exhibited enhanced growth at 1 M NaCl compared to salt-free conditions, at 29 °C. Two different populations (3AcDON and 15AcDON) of *F. pseudograminearum* showed a strong tolerance to salt stress (1 M NaCl) under simulated laboratory conditions [35].

Conversely, mycotoxin production was dramatically reduced or even completely inhibited in the presence of salt. All isolates produced ZEA under all tested conditions, with optimal production observed in the absence of salt at 22 and 25 °C (Table 1). Interestingly, in isolates 3B and CBS 109956, the inhibitory effects of low NaCl concentrations were mitigated at 22 °C, as ZEA production did not significantly differ from the salt-free condition. However, ZEA biosynthesis was markedly suppressed at 28 °C across all treatments (Table 1).

Deoxynivalenol production varied significantly among the isolates. Isolates PVS-Fu 7 and ColPat-351 did not produce DON under any of the tested conditions. In contrast, isolate 3B was the highest producer, with optimal DON synthesis occurring at 25 °C in the absence of NaCl, exceeding 1000 µg kg^−1^. The presence of salt led to a reduction in DON biosynthesis, which was completely inhibited outside the optimal production conditions (Table 2). Isolate CBS 109956 invariably produced small amounts of DON and was highly sensitive to salinity. In both 3B and CBS 109956 isolates, DON production was suppressed at 28 °C across all salinity levels and all temperatures (Table 2). Overall, isolate 3B displayed the strongest mycotoxigenic ability and highest tolerance to salt stress (Table 1 and Table 2).

These findings are consistent with those of a previous study in which the ability of *F. pseudograminearum* isolates 3B, PVS-Fu 7, and ColPat-351 to produce ZEA and DON was investigated [29].

Zearalenone production was detected in all isolates, although with variable yields. In contrast, DON levels were below LOD in isolates PVS-Fu 7 and ColPat-351, supporting their classification as non- or low-DON producers. This is consistent with previous findings by Delli Compagni et al. [29] and Cui et al. [25]. The latter authors reported that the optimal temperature for ZEA and DON production in *F. pseudograminearum* was 25 °C, with detectable levels also observed at 20 and 30 °C [25], and reduced water activity (a_w_) significantly inhibited mycotoxin biosynthesis, supporting our observation that salinization of the substrate suppresses mycotoxin production.

In our study, ZEA production was observed at sub- or supra-optimal temperatures (22 and 28 °C), while DON was not detected at 28 °C. Conversely, biosynthesis of both ZEA and DON by *F. pseudograminearum* was observed by Blaney and Dodman [44] at 28 °C and by Clear et al. [45] at 23 °C, although with considerable variability among isolates tested in both studies.

Our work also demonstrated that different *F. pseudograminearum* isolates showed distinct mycotoxigenic abilities. The production of mycotoxin by *Fusarium* species is known to be isolate-specific as previously reported by many authors [20,45,46,47,48,49].

The most effective strategy to minimize mycotoxin contamination is to limit fungal growth. However, favorable conditions for fungal proliferation do not always correlate with increasing mycotoxin production [9,50]. Although NaCl supplementation enhanced the growth rate of all *F. pseudograminearum* isolates (Figure 1 a–d), it concurrently reduced mycotoxigenic potential (Table 1 and Table 2). Although salinity may create environmental conditions that support or even stimulate fungal growth, the associated reduction in a_w_ can become a limiting factor for mycotoxin biosynthesis. Under our experimental conditions, the calculated a_w_ values of the culture media decreased from 0.998 in PDA without salt to 0.982 in PDA supplemented with 28 g L^−1^ NaCl. This trend is consistent with the well-established role of a_w_ as a key regulator of secondary metabolism, where even relatively small decreases in a_w_ can markedly affect toxin biosynthesis [51]. Understanding both the optimal and marginal a_w_ × T conditions for mycotoxigenic fungi is essential for assessing the relative risks of toxin contamination [52].

Moreover, salinity can negatively impact mycotoxin production through direct interference with biosynthetic pathways and osmotic stress. The production of trichothecenes in *F. graminearum* was markedly inhibited by NaCl, which had no obvious effect on fungal growth. The involvement of kinases (MAPKs) of the two-component osmotic signal transduction pathway negatively regulated trichothecene production [53]. In addition, high salt levels can downregulate *Tri* genes involved in the biosynthetic pathways of trichothecenes, directly preventing toxin formation [54].

On the other hand, it is well established that fungi adapt to salinity stress by accumulating organic osmolytes (such as proline, sugars and non-cyclic polyols) [32]. This adaptive process is energetically demanding, and the associated metabolic costs may divert resources away from other physiological functions [55].

From a phytopathological and agronomical perspective, Smith et al. [56] reported that crown rot symptoms caused by *F*. *pseudograminearum* were exacerbated in saline soils, a finding that carries important practical implications for both researchers and grain producers. The fitness of *F. pseudograminearum* during stem base colonization has been shown to correlate with the level of DON production [57], and several studies have demonstrated a role for DON in pathogen virulence [4,58].

Finally, the intracellular level of K^+^ and Na^+^ within hyphae was measured under varying salinity and temperature conditions. In all isolates, increasing NaCl concentrations led to a corresponding rise in Na^+^ levels within the hyphae.

Even without the addition of NaCl to the culture medium, all fungal isolates exhibited a baseline level of intracellular Na^+^, with isolates 3B and CBS 109956 showing the highest concentration (Figure 2a,b). This basal Na^+^ level significantly decreased at 28 °C across all isolates. However, at a NaCl concentration of 7 g L^−1^, the effect of temperature on Na^+^ accumulation was less pronounced (Figure 2a–d). While all isolates exhibited increased Na^+^ levels under low salinity, isolates 3B and CBS 109956 showed a less pronounced response when compared to their baseline levels in salt-free conditions.

Under maximum salinity conditions, fungal Na^+^ accumulation was temperature-dependent. While isolate 3B experienced a significant reduction in hyphal Na^+^ at 28 °C (Figure 2a), the remaining isolates demonstrated a consistent increase in Na^+^ levels with rising temperature (Figure 2b–d). The notably high Na^+^ concentration observed in isolate 3B aligns with its origin from *S. europaea*, a halophyte commonly found in saline habitats, suggesting a possible physiological adaptation to high-salinity conditions [29]. Isolate 3B demonstrated a marked increase in hyphal K^+^ concentration under saline conditions, with K^+^ levels rising in parallel with NaCl concentration (Figure 2a). Notably, all isolates except ColPat-351 exhibited a peak in K^+^ accumulation at 28 g L^−1^, indicating a potential mechanism for concurrent Na^+^ uptake and K^+^ retention within the hyphae (Figure 2a–c).

An increased passive efflux of K^+^ from hyphae is likely a major factor contributing to growth inhibition in fungi exposed to elevated Na^+^ concentrations in the medium [59]. In parallel, many fungi possess the ability to efficiently absorb Na^+^ from their surroundings. High-affinity, high-rate Na^+^ transporters, capable of mediating the uptake of either K^+^ or Na^+^, were identified in certain fungal species. However, this trait is not universally present across all fungi; putative genes encoding such transporters have also been reported in *Fusarium sporotrichioides* [60]. In addition, functional Na^+^ transporters have been characterized in the fungus *Magnaporthe oryzae*, leading to increased intracellular Na^+^ levels following exposure to NaCl [61]. Consistently, the K^+^/Na^+^ molar ratio progressively decreased under saline conditions (Table S9). This trend appears to be primarily driven by the isolates ability to accumulate Na^+^ in response to NaCl, rather than by a significant loss of K^+^, as K^+^ levels remained relatively stable across salinity levels, particularly in isolates 3B and CBS 109956 (Figure 2a,b).

To our knowledge, no quantitative studies have been conducted on the ability of *Fusarium* spp. to absorb Na^+^ from the culture medium, making direct comparisons with our measurements unfeasible. Previous research on the effects of NaCl on *Fusarium* spp. has highlighted alterations in the lipid and polysaccharide composition of fungal cells [32]. Therefore, further investigations are needed to elucidate the biochemical mechanisms underlying the response of *F. pseudograminearum* to salinity, to better understand its adaptive strategies, and to assess its environmental fitness as a pathogenic fungus.

## 5. Conclusions

This study demonstrates that *F. pseudograminearum* is well adapted to saline conditions and supports its classification as a weak halophilic fungus. All tested isolates exhibited enhanced growth in the presence of NaCl and accumulated Na^+^ within hyphae as salinity increased while maintaining intracellular K^+^ levels. Furthermore, 28 °C appears to represent a threshold temperature for mycotoxigenic activity, as demonstrated by the marked inhibition of ZEA and DON production at this temperature. Overall, these findings provide valuable insights into the physiological responses of *F. pseudograminearum* to NaCl salinity and temperature. The stimulatory effect of salinity on mycelial growth may reflect an adaptive strategy for survival in saline conditions; however, the metabolic cost of coping with osmotic stress likely diverts resources away from secondary metabolism. Consequently, environments characterized by high salinity may support fungal colonization without necessarily increasing the risk of mycotoxin contamination. The results of this study and those reported in the literature suggest that salinity exerts a dual effect on *F. pseudograminearum*: on the one hand, it may help reduce mycotoxin production, while on the other, it can exacerbate disease severity. These findings underscore the importance of considering both growth and mycotoxin production dynamics when assessing the ecological and pathological implications of salinity in plant-growing systems. Since earlier assessments of disease severity in the *F. pseudograminearum*–*S. europaea* pathosystem did not incorporate salt stress, upcoming work will investigate how salt stress influences the virulence of *F. pseudograminearum* towards this plant species. Understanding the adaptive mechanisms of plant pathogens is crucial for developing effective and sustainable disease management strategies. The information presented in this work may contribute to identifying environmental scenarios in which the pathogen can survive without producing hazardous levels of mycotoxins, and to designing agronomic interventions that maintain conditions unfavorable for mycotoxin synthesis, even when the pathogen is present. This aspect becomes even more significant when considering that the cultivation of halophytic plants is increasingly recognized as a sustainable agricultural approach, driven by the escalating severity of soil and water salinization, a phenomenon that poses a major challenge to global food security and crop productivity.

## Figures and Tables

**Table 1:** Production of zearalenone (ZEA) by four *Fusarium pseudograminearum* isolates (3B, CBS 109956, PVS-Fu 7, and ColPat-351) under different temperature and salinity conditions. Data were natural log (*ln*) transformed and subjected to two-way ANOVA. Means (*n* = 3; ±SE) were separated using Tukey’s HSD post hoc test. Different letters within each isolate indicate statistically significant differences at *p* ≤ 0.01 or *p* ≤ 0.001.

Isolate	Temperature (°C)	ZEA (mg kg^−1^ Dry Weight)					
		Salinity (NaCl g L^−1^)					
		0		7		28	
3B	22	143 ± 18.0	c	126.6 ± 12.2	c	11.0 ± 3.5	d
	25	1143.1 ± 94.9	a	348.8 ± 13.8	b	126.4 ± 2.8	c
	28	3.9 ± 1.0	d	0.8 ± 0.4	d	0.3 ± 0.1	d
CBS 109956	22	114.8 ± 3.8	bc	124.4 ± 4.0	b	3.0 ± 0.1	d
	25	180.6 ± 30.9	a	63.6 ± 2.5	c	4.56 ± 0.9	d
	28	7.5 ± 0.5	d	4.5 ± 0.8	d	0.6 ± 0.1	d
PVS-Fu 7	22	120.5 ± 43.5	a	13.1 ± 1.2	c	3.2 ± 0.4	c
	25	114.2 ± 31.9	ab	38.3 ± 13.8	bc	0.5 ± 0.1	c
	28	24.6 ± 1.4	bc	2.3 ± 0.2	c	0.3 ± 0.1	c
ColPat-351	22	59.4 ± 4.5	a	15.5 ± 3.7	bc	1.3 ± 0.1	d
	25	22.4 ± 1.5	b	4.8 ± 0.1	d	1.5 ± 0.1	d
	28	7.0 ± 1.7	cd	3.7 ± 0.4	d	0.5 ± 0.1	d

**Table 2:** Production of deoxynivalenol (DON) by two *Fusarium pseudograminearum* isolates (3B and CBS 109956) under different temperature and salinity conditions. Data were natural log (*ln*) transformed and subjected to two-way ANOVA. Means (*n* = 3; ±SE) were separated by Tukey’s HSD post hoc test. Different letters within each isolate indicate statistically significant differences at *p* ≤ 0.01 or *p* ≤ 0.001.

Isolate	Temperature (°C)	DON (µg kg^−1^ Dry Weight)				
		Salinity (NaCl g L^−1^)				
		0		7		28
3B	22	41 ± 5	c	<LOD *		<LOD *
	25	1640 ± 35	a	725 ± 33	b	<LOD *
	28	<LOD *		<LOD *		<LOD *
CBS 109956	22	60 ± 7	a	<LOD *		<LOD *
	25	72 ± 27	a	<LOD *		<LOD *
	28	<LOD *		<LOD *		<LOD *

* LOD: limit of detection.

## Data Availability

All data generated or analyzed during this study are included in this publication.

## Supplementary Materials

The following supporting information can be downloaded at: https://www.mdpi.com/article/10.3390/biology15030280/s1, Table S1. Three-way ANOVA results for the daily growth rate of the four *Fusarium pseudograminearum* isolates; Table S2. Two-way ANOVA results for the daily growth rate of the four *Fusarium pseudograminearum* isolates; Table S3. Three-way ANOVA results for zearalenone (ZEA) production by the four *Fusarium pseudograminearum* isolates; Table S4. Two-way ANOVA results for zearalenone (ZEA) production by the four *Fusarium pseudograminearum* isolates; Table S5. Three-way ANOVA results for deoxynivalenol (DON) production by two *Fusarium pseudograminearum* isolates (3B and CBS 109956); Table S6. Two-way ANOVA results for deoxynivalenol (DON) production by two *Fusarium pseudograminearum* isolates (3B and CBS 109956); Table S7. Three-way ANOVA results for K^+^ concentration (mg g^−1^) within the hyphae of the four *Fusarium pseudograminearum* isolates; Table S8. Three-way ANOVA results for Na^+^ concentration (mg g^−1^) within the hyphae of the four *Fusarium pseudograminearum* isolates; Table S9. Three-way ANOVA results for K^+^/Na^+^ molar ratio within the hyphae of the four *Fusarium pseudograminearum* isolates; Table S10. Two-way ANOVA results for K^+^ concentration (mg g^−1^) within the hyphae of the four *Fusarium pseudograminearum* isolates; Table S11. Three-way ANOVA results for Na^+^ concentration (mg g^−1^) within the hyphae of the four *Fusarium pseudograminearum* isolates.
