# Supplementary material for: Fusarium pseudograminearum Isolates Show Enhanced Growth and Na+ Uptake but Suppressed Mycotoxin Production After Exposure to NaCl at Different Temperatures
Source: Biology (Basel). 2026 Feb 4;15(3):280. doi: 10.3390/biology15030280 (PMC12897232; doi:10.3390/biology15030280)
Supplement: Supplementary file 1 [file biology-15-00280-s001.zip › biology-4098556-supplementary.pdf]

Supplementary Materials

Fusarium pseudograminearum isolates show enhanced growth and Na^+ uptake but suppressed mycotoxin production after exposure to NaCl at different temperatures

Emiliano Delli Compagni [1,*], Mario Masiello [2], Miriam Haidukowski [2], Giulia Carmassi [1], Antonio Moretti [2], Alberto Pardossi [1,3], and Susanna Pecchia [1,3,*]

[1] Department of Agriculture Food and Environment, University of Pisa, Via del Borghetto 80, 56124 Pisa, Italy; G.C. giulia.carmassi@unipi.it; A.P. alberto.pardossi@unipi.it

[2] Institute of Sciences of Food Production, National Research Council– CNR-ISPA, Via Amendola 122/O, 70126 Bari, Italy; M.M. mario.masiello@cnr.it; M.H. edithmiriam.haidukowski@cnr.it; A.M. antonio.moretti@cnr.it

[3] Interdepartmental Research Center Nutrafood "Nutraceuticals and Food for Health", University of Pisa, Via del Borghetto 80, 56124 Pisa, Italy

* Correspondence: Emiliano Delli Compagni emiliano.dellicompagni@phd.unipi.it; Susanna Pecchia susanna.pecchia@unipi.it

Table S1. Three-way ANOVA results for the daily growth rate of the four *Fusarium pseudograminearum* isolates (3B, CBS 109956, PVS-Fu 7, and ColPat-351). The ability of the isolates to grow in response to salinity and temperature was tested on Potato Dextrose Agar (PDA). The isolates were grown at 10, 15, 20, 25, and 30 °C and at 0, 7, 14, 21, and 28 g L^{-1} NaCl, and the daily growth rate (mm day^{-1}) was recorded. The experiment consisted of five biological replicates for each temperature and salinity. Means (n = 5; ± SE) were separated by Tukey's HSD post-hoc test.

Effect Tests

Factor	Degrees of freedom	Sum of squares	Mean sum of squares	F ratio	*p*
Isolate	3	913	304.4	686,124	≤0.001
Temperature	4	6113	1528.2	3,444,405	≤0.001
Salinity	4	535	133.6	301,212	≤0.001
Isolate x Temperature	12	750	62.5	140,950	≤0.001
Isolate x Salinity	12	83	6.9	15,527	≤0.001
Temperature x Salinity	16	249	15.6	35,138	≤0.001
Isolate x Temperature x Salinity	48	128	2.7	6,015	≤0.001
Residuals	400	177	0.4		

LSMeans Differences Tukey HSD (Main effects)

Factor	Level	Daily growth rate (mm day^{-1})
Isolate	3B	11.0 ± 0.3 a
	CBS 109956	7.7 ± 0.1 c
	PVS-Fu 7	10.9 ± 0.4 a
	ColPat-351	9.1 ± 0.3 b
Temperature (°C)	10	3.7 ± 0.0 e
	15	8.3 ± 0.1 d
	20	11.5 ± 0.2 b
	25	14.0 ± 0.3 a
	30	10.7 ± 0.3 c
Salinity (g L^{-1} NaCl)	0	7.7 ± 0.3 d
	7	10.4 ± 0.4 a
	14	10.5 ± 0.4 a
	21	10.1 ± 0.4 b
	28	9.5 ± 0.4 c

Table S2. Two-way ANOVA results for the daily growth rate of the four *Fusarium pseudograminearum* isolates (3B, CBS 109956, PVS-Fu 7, and ColPat-351). The ability of the isolates to grow in response to salinity and temperature was tested on PDA plates. The isolates were grown at 10, 15, 20, 25, and 30 °C and at 0, 7, 14, 21, and 28 g L^{-1} NaCl, and the daily growth rate (mm day^{-1}) was recorded. The experiment consisted of five biological replicates for each temperature and salinity.

Factor	Degrees of freedom	Sum of squares	Mean sum of squares	F ratio	*p*
F. pseudograminearum 3B					
Temperature	4	1909.9	477.5	2740.8	≤0.001
Salinity	4	169.5	42.4	243.3	≤0.001
Temperature x Salinity	16	64.9	4.1	23.3	≤0.001
Residuals	100	17.4	0.2		
F. pseudograminearum CBS 109956					
Temperature	4	491.5	122.9	969.3	≤0.001
Salinity	4	28.5	7.0	55.6	≤0.001
Temperature x Salinity	16	52.2	3.3	25.7	≤0.001
Residuals	100	12.7	0.1		
F. pseudograminearum PVS-Fu 7					
Temperature	4	2516.4	629.1	1563.8	≤0.001
Salinity	4	143.2	35.8	88.9	≤0.001
Temperature x Salinity	16	196.8	12.3	30.6	≤0.001
Residuals	100	40.2	0.4		
F. pseudograminearum ColPat-351					
Temperature	4	1945.7	486.4	453.9	≤0.001
Salinity	4	276.3	69.1	64.5	≤0.001
Temperature x Salinity	16	63.7	4.0	3.7	≤0.001
Residuals	100	107.2	1.1		

Table S3. Three-way ANOVA results for zearalenone (ZEA) production by the four *Fusarium pseudograminearum* isolates (3B, CBS 109956, PVS-Fu 7, and ColPat-351). Data were *ln* transformed to meet ANOVA assumptions. ZEA production was evaluated by growing the isolates on moistened barley grains for 21 days at temperatures of 22, 25, and 28 °C and at NaCl concentrations of 0, 7, and 28 g L^{-1}. The experiment consisted of three biological replicates for each temperature and salinity level. Means (n = 3; ± SE) were separated by Tukey's HSD post-hoc test.

Effect Tests

Factor	Degrees of freedom	Sum of squares	Mean sum of squares	F ratio	*p*
Isolate	3	39.9	13.3	68,472	≤0.001
Temperature	2	171.7	85.8	442,088	≤0.001
Salinity	2	200.6	100.3	516,484	≤0.001
Isolate x Temperature	6	80.4	13.4	68,988	≤0.001
Isolate x Salinity	6	19.1	3.2	16,398	≤0.001
Temperature x Salinity	4	3.8	0.9	4,885	≤0.010
Isolate x Temperature x Salinity	12	20.0	1.7	8,605	≤0.001
Residuals	72	14.0	0.2		

LSMeans Differences Tukey HSD (Main effects)

Factor	Level	ZEA (mg kg^{-1} of dry weight)
Isolate	3B	211.5 ± 68.4 a
	CBS 109956	55.9 ± 12.9 b
	PVS-Fu 7	35.3 ± 10.2 c
	ColPat-351	12.8 ± 3.5 d
Temperature (°C)	22	61.3 ± 10.1 a
	25	170.8 ± 52.7 a
	28	4.6 ± 1.1 b
Salinity (g L^{-1} NaCl)	0	161.4 ± 51.6 a
	7	62.5 ± 16.4 b
	28	12.7 ± 5.8 c

Table S4. Two-way ANOVA results for zearalenone (ZEA) production by the four *Fusarium pseudograminearum* isolates (3B, CBS 109956, PVS-Fu 7, and ColPat-351). Data were *ln* transformed to meet ANOVA assumptions. ZEA production was evaluated by growing the isolates on moistened barley grains for 21 days at temperatures of 22, 25, and 28 °C and at NaCl concentrations of 0, 7, and 28 g L^{-1}. The experiment consisted of three biological replicates for each temperature and salinity level.

Factor	Degrees of freedom	Sum of squares	Mean sum of squares	F ratio	*p*
F. pseudograminearum 3B					
Temperature	2	185.5	92.8	229.6	≤0.001
Salinity	2	25.6	12.8	31.7	≤0.001
Temperature x Salinity	4	8.9	2.2	5.5	≤0.010
Residuals	18	7.3	0.4		
F. pseudograminearum CBS109956					
Temperature	2	40.6	20.3	398.3	≤0.001
Salinity	2	57.0	28.5	559.5	≤0.001
Temperature x Salinity	4	2.9	0.7	14.1	≤0.001
Residuals	18	0.9	0.05		
F. pseudograminearum PVS-Fu 7					
Temperature	2	17.1	8.6	40.9	≤0.001
Salinity	2	86.7	43.4	207.0	≤0.001
Temperature x Salinity	4	6.7	1.7	7.9	≤0.001
Residuals	18	3.8	0.2		
F. pseudograminearum ColPat-351					
Temperature	2	10.3	5.1	84.7	≤0.001
Salinity	2	42.9	21.4	353.6	≤0.001
Temperature x Salinity	4	2.3	0.6	9.4	≤0.001
Residuals	18	1.1	0.1		

Table S5. Three-way ANOVA results for deoxynivalenol (DON) production by the two *Fusarium pseudograminearum* isolates (3B and CBS 109956). Since isolates PVS-Fu 7 and ColPat-351 did not produce the mycotoxin, they were excluded from the analysis. Data were *ln* transformed to meet ANOVA assumptions. Where no mycotoxin was detected, data were replaced with LOD/2 values for statistical analysis. DON production was evaluated by growing the isolates on moistened barley grains for 21 days at temperatures of 22, 25, and 28 °C and at NaCl concentrations of 0, 7, and 28 g L^{-1}. The experiment consisted of three biological replicates for each temperature and salinity level. Means (n = 3; ± SE) were separated by Tukey's HSD post-hoc test.

Effect Tests

**Factor**	**Degrees of freedom**	**Sum of squares**	**Mean sum of squares**	**F ratio**	***p***
Isolate	1	10.4	10.4	384.7	≤0.001
Temperature	2	45.7	22.8	846.9	≤0.001
Salinity	2	42.0	21.0	779.6	≤0.001
Isolate x Temperature	2	23.6	11.8	437.6	≤0.001
Isolate x Salinity	2	6.2	3.1	115.8	≤0.001
Temperature x Salinity	4	31.1	7.8	288.7	≤0.001
Isolate x Temperature x Salinity	4	13.0	3.3	120.8	≤0.001
Residuals	36	1.0.	0.03		

LSMeans Differences Tukey HSD (Main effects)

**Factor**	**Level**	**DON (μg kg^{-1} of dry weight)**
Isolate	3B	270.3 ± 104.5 a
	CBS 109956	18.4 ± 5.6 b
Temperature (°C)	22	19.9 ± 5.4 b
	25	408.2 ± 147.5 a
	28	<LOD c
Salinity (g L^{-1} NaCl)	0	303.2 ± 145 a
	7	124.9 ± 65.2 b
	28	<LOD c

Table S6. Two-way ANOVA results for deoxynivalenol (DON) production by two *Fusarium pseudograminearum* isolates (3B and CBS 109956). Since isolates PVS-Fu 7 and ColPat-351 did not produce the mycotoxin, they were excluded from the analysis. Data were *ln* transformed to meet ANOVA assumptions. Where no mycotoxin was detected, data were replaced with LOD/2 values for statistical analysis. DON production was evaluated by growing the isolates on moistened barley grains for 21 days at temperatures of 22, 25, and 28 °C and at NaCl concentrations of 0, 7, and 28 g L^{-1}. The experiment consisted of three biological replicates for each temperature and salinity level.

**Factor**	**Degrees of freedom**	**Sum of squares**	**Mean sum of squares**	**F ratio**	***p***
F. pseudograminearum 3B					
Temperature	2	65.2	32.6	35741	≤0.001
Salinity	2	31.8	15.9	17432	≤0.001
Temperature x Salinity	4	35.9	9.0	9850	≤0.001
Residuals	18	0.02	0.0		
F. pseudograminearum CBS109956					
Temperature	2	4.4	2.2	48.0	≤0.001
Salinity	2	17.3	8.7	192.1	≤0.010
Temperature x Salinity	4	8.7	2.2	48.0	≤0.010
Residuals	18	0.8	0.05		

Table S7. Three-way ANOVA results for K^+ concentration (mg g^{-1} of dry weight) within the hyphae of the four *Fusarium pseudograminearum* isolates (3B, CBS 109956, PVS-Fu 7, and ColPat-351) grown at 22, 25, and 28 °C on Potato Dextrose Agar (PDA) plates amended with 0, 7, and 28 g L^{-1} NaCl for one week. The experiment consisted of three biological replicates for each temperature and salinity level. Means (n = 3; ± SE) were separated by Tukey's HSD post-hoc test.

Effect Tests

Factor	Degrees of freedom	Sum of squares	Mean sum of squares	F ratio	*p*
Isolate	3	1166.5	388.8	612,646	≤0.001
Temperature	2	3.1	1.6	2,447	0.094
Salinity	2	33.3	16.7	26,202	≤0.001
Isolate x Temperature	6	101.5	16.9	26,655	≤0.001
Isolate x Salinity	6	86.9	14.5	22,814	≤0.001
Temperature x Salinity	4	13.0	3.3	5,115	≤0.010
Isolate x Temperature x Salinity	12	60.0	5.0	7,879	≤0.001
Residuals	72	45.7	0.6		

LSMeans Differences Tukey HSD (Main effects)

Factor	Level	K^+ (mg g^{-1} of dry weight)
Isolate	3B	15.4 ± 0.3 a
	CBS 109956	15.8 ± 0.4 a
	PVS-Fu 7	9.5 ± 0.3 b
	ColPat-351	8.6 ± 0.2 c
Temperature (°C)	22	12.3 ± 0.5 a
	25	12.5 ± 0.5 a
	28	12.1 ± 0.7 a
Salinity (g L^{-1} NaCl)	0	12.1 ± 0.4 b
	7	11.7 ± 0.6 b
	28	13.1 ± 0.7 a

Table S8. Three-way ANOVA results for Na^+ concentration (mg g^{-1} of dry weight) within the hyphae of the four *Fusarium pseudograminearum* isolates (3B, CBS 109956, PVS-Fu 7, and ColPat-351) grown at 22, 25, and 28 °C on Potato Dextrose Agar (PDA) plates amended with 0, 7, and 28 g L^{-1} NaCl for one week. The experiment consisted of three biological replicates for each temperature and salinity level. Means (n = 3; ± SE) were separated by Tukey's HSD post-hoc test.

Effect Tests

**Factor**	**Degrees of freedom**	**Sum of squares**	**Mean sum of squares**	**F ratio**	***p***
Isolate	3	300.0	100.1	486.6	≤0.001
Temperature	2	10.0	5.1	24.6	≤0.001
Salinity	2	4550.0	2275.0	11,054.9	≤0.001
Isolate x Temperature	6	50.0	8.3	40.3	≤0.001
Isolate x Salinity	6	80.0	13.3	64.8	≤0.001
Temperature x Salinity	4	84.0	21.0	102.1	≤0.001
Isolate x Temperature x Salinity	12	53.0	4.4	21.4	≤0.001
Residuals	72	15.0	0.2		

LSMeans Differences Tukey HSD (Main effects)

**Factor**	**Level**	**Na^+ (mg g^{-1} of dry weight)**
Isolate	3B	11.6 ± 1.4 a
	CBS 109956	10.5 ± 1.3 b
	PVS-Fu 7	7.7 ± 1.2 c
	ColPat-351	7.8 ± 1.2 c
Temperature (°C)	22	9.0 ± 1.0 b
	25	9.6 ± 1.1 a
	28	9.6 ± 1.3 a
Salinity (g L^{-1} NaCl)	0	2.7 ± 0.3 c
	7	7.3 ± 0.1 b
	28	18.2 ± 0.5 a

Table S9. Three-way ANOVA results for the K^+/Na^+ molar ratio within the hyphae of the four *Fusarium pseudograminearum* isolates (3B, CBS 109956, PVS-Fu 7, and ColPat-351) grown at 22, 25, and 28 °C on Potato Dextrose Agar (PDA) plates amended with 0, 7, and 28 g L^{-1} NaCl for one week. The experiment consisted of three biological replicates for each temperature and salinity level. Means (n = 3; ± SE) were separated by Tukey's HSD post-hoc test.

Effect Tests

Factor	Degrees of freedom	Sum of squares	Mean sum of squares	F ratio	*p*
Isolate	3	67.9	22.6	72.4	≤0.001
Temperature	2	52.9	26.5	84.6	≤0.001
Salinity	2	409.3	204.7	653.9	≤0.001
Isolate x Temperature	6	23.3	3.9	12.4	≤0.001
Isolate x Salinity	6	181.6	30.3	96.7	≤0.001
Temperature x Salinity	4	113.3	28.3	90.5	≤0.001
Isolate x Temperature x Salinity	12	56.6	4.7	15.1	≤0.001
Residuals	72	22.5	0.3		

LSMeans Differences Tukey HSD (Main effects)

Factor	Level	K^+/Na^+ molar ratio
Isolate	3B	1.1 ± 0.1 c
	CBS 109956	1.4 ± 0.2 c
	PVS-Fu 7	3.0 ± 0.8 a
	ColPat-351	2.5 ± 0.6 b
Temperature (°C)	22	1.5 ± 0.2 b
	25	1.5 ± 0.2 b
	28	3.0 ± 0.7 a
Salinity (g L^{-1} NaCl)	0	4.7 ± 0.6 a
	7	0.9 ± 0.0 b
	28	0.4 ± 0.0 c

Table S10. Two-way ANOVA results for Na^{+} concentration (mg g^{-1} of dry weight) within the hyphae of the four *Fusarium pseudograminearum* isolates (3B, CBS 109956, PVS-Fu 7, and ColPat-351) grown at 22, 25, and 28 °C on Potato Dextrose Agar (PDA) plates amended with 0, 7, and 28 g L^{-1} NaCl for one week. The experiment consisted of three biological replicates for each temperature and salinity level.

Factor	Degrees of freedom	Sum of squares	Mean sum of squares	F ratio	*p*
F. pseudograminearum 3B					
Temperature	2	24.1	12.0	28.3	≤0.001
Salinity	2	1415.5	707.7	1662.6	≤0.001
Temperature x Salinity	4	7.2	1.8	4.2	≤0.050
Residuals	18	7.7	0.4		
F. pseudograminearum CBS109956					
Temperature	2	10.4	5.2	34.2	≤0.001
Salinity	2	1250.6	625.3	4115.3	≤0.001
Temperature x Salinity	4	52.6	13.2	86.6	≤0.001
Residuals	18	2.7	0.2		
F. pseudograminearum PVS-Fu 7					
Temperature	2	19.7	9.9	122.5	≤0.001
Salinity	2	956.2	478.1	5934.5	≤0.001
Temperature x Salinity	4	45.0	11.2	139.5	≤0.001
Residuals	18	1.5	0.1		
F. pseudograminearum ColPat-351					
Temperature	2	5.6	2.8	17.1	≤0.001
Salinity	2	1007.5	503.8	3054	≤0.001
Temperature x Salinity	4	32.2	8.0	48.8	≤0.001
Residuals	18	3.0	0.2		

Table S11. Two-way ANOVA results for K^+ concentration (mg g^{-1} of dry weight) within the hyphae of the four *Fusarium pseudograminearum* isolates (3B, CBS 109956, PVS-Fu 7, and ColPat-351) grown at 22, 25, and 28 °C on Potato Dextrose Agar (PDA) plates amended with 0, 7, and 28 g L^{-1} NaCl for one week. The experiment consisted of three biological replicates for each temperature and salinity level.

Factor	Degrees of freedom	Sum of squares	Mean sum of squares	F ratio	*p*
F. pseudograminearum 3B					
Temperature	2	5.4	2.7	5.5	≤0.050
Salinity	2	50.0	25.0	51.4	≤0.001
Temperature x Salinity	4	6.0	1.5	3.0	≤0.010
Residuals	18	8.8	0.5		
F. pseudograminearum CBS109956					
Temperature	2	34.3	17.2	14.0	≤0.001
Salinity	2	43.7	21.8	17.8	≤0.001
Temperature x Salinity	4	15.0	3.8	3.0	≤0.050
Residuals	18	22.1	1.2		
F. pseudograminearum PVS-Fu 7					
Temperature	2	61.8	30.9	54.0	≤0.001
Salinity	2	13.7	6.9	12.0	≤0.001
Temperature x Salinity	4	14.0	3.5	6.1	≤0.010
Residuals	18	10.3	0.6		
F. pseudograminearum ColPat-351					
Temperature	2	3.1	1.6	6.2	≤0.010
Salinity	2	12.7	6.4	25.2	≤0.001
Temperature x Salinity	4	38.0	9.5	37.7	≤0.001
Residuals	18	4.5	0.2		

Disclaimer/Publisher's Note: The statements, opinions and data contained in all publications are solely those of the individual author(s) and contributor(s) and not of MDPI and/or the editor(s). MDPI and/or the editor(s) disclaim responsibility for any injury to people or property resulting from any ideas, methods, instructions or products referred to in the content.
